# Ultrafiltered recombinant AAV8 vector can be safely administered *in vivo* and efficiently transduces liver

**DOI:** 10.1371/journal.pone.0194728

**Published:** 2018-04-05

**Authors:** Mark D. Kleven, Michelle M. Gomes, Aaron M. Wortham, Caroline A. Enns, Christoph A. Kahl

**Affiliations:** 1 Department of Cell, Developmental & Cancer Biology, Oregon Health & Science University, Portland, Oregon, United States of America; 2 Oregon National Primate Research Center, Oregon Health & Science University, Portland, Oregon, United States of America; Justus Liebig Universitat Giessen, GERMANY

## Abstract

Viral vectors are extensively purified for use in biomedical research, in order to separate biologically active virus particles and to eliminate production related impurities that are assumed to be detrimental to the host. For recombinant adeno-associated virus (rAAV) vectors this is typically accomplished using density gradient-based methods, which are tedious and require specialized ultracentrifugation equipment. In order to streamline the preparation of rAAV vectors for pilot and small animal studies, we recently devised a simple ultrafiltration approach that permits rapid virus concentration and partial removal of production-related impurities. Here we show that systemic administration of such rapidly prepared (RP) rAAV8 vectors in mice is safe and efficiently transduces the liver. Across a range of doses, delivery of RP rAAV8-CMV-eGFP vector induced enhanced green fluorescent protein (eGFP) expression in liver that was comparable to that obtained from a conventional iodixanol gradient-purified (IP) vector. Surprisingly, no liver inflammation or systemic cytokine induction was detected in RP rAAV injected animals, revealing that residual impurities in the viral vector preparation are not deleterious to the host. Together, these data demonstrate that partially purified rAAV vector can be safely and effectively administered *in vivo*. The speed and versatility of the RP method and lack of need for cumbersome density gradients or expensive ultracentrifuge equipment will enable more widespread use of RP prepared rAAV vectors, such as for pilot liver gene transfer studies.

## Introduction

Recombinant adeno-associated virus (rAAV) vectors are versatile tools for gene therapy due to their ability to mediate transgene expression for an extended period, their low immunogenicity profile and their wide tissue tropism. They are based on adeno-associated virus (AAV), which is a small, single-stranded DNA virus belonging to the *Parvoviridae* family that can infect both dividing and non-dividing cells, but which requires a helper virus to replicate and is not known to cause any disease in humans [1]. rAAV vectors consist of a recombinant DNA genome that lacks the viral coding sequences and instead encodes the desired transgene sequences. The tissue tropism of rAAV vectors can be altered by packaging the genome into capsid shells from AAV serotypes that use different cellular receptors. rAAV vectors are commonly produced in human embryonic kidney (HEK) 293 cell lines by triple transfection of (1) a plasmid carrying transgene sequences flanked by viral inverted terminal repeats (ITR), (2) the packaging plasmid providing replication (rep) and encapsidation (cap) genes of the desired AAV serotype *in trans*, and (3) a helper plasmid containing the adenovirus genes E2a, E4, and VA RNAs, which, together with the adenovirus E1 gene expressed in HEK producer cells, enable viral replication [2]. Viral particles are typically harvested directly from producer cells and/or the culture media several days post-transfection. Purification of rAAV vector from the crude cellular lysate typically involves setting up cesium chloride or iodixanol density gradients, which permit efficient enrichment of genome-containing virus particles away from cellular debris. However, these purification methods are time and labor-intensive, require specialized laboratory expertise, and lead to virus loss, at best recovering ~50% of the initial viral genome-containing particles [3].

The liver is a major metabolic organ and the expression of genes in the liver is an invaluable tool for metabolic studies and gene therapy. rAAV vectors of serotype 8 (AAV8) efficiently transduce muscle, liver, and brain tissues [4]^,^[5]. Furthermore, rAAV8 shows a 10–100-fold improved efficiency of liver gene transfer compared to rAAV2 [6], with minimal lag between vector administration and gene expression [4]. In the present study we wanted to evaluate if rAAV8 vector prepared by a simple ultrafiltration approach and injected in an animal host could efficiently enable gene transfer to the liver. To this end, we compared the transduction efficiency, safety profile, and immunogenicity of standard iodixanol purified (IP) rAAV8 vector with rapidly prepared (RP) rAAV8 vector following intraperitoneal administration in adult mice. Despite significant production-related impurities, the RP-AAV8 vector showed comparable levels of liver transgene expression as the conventionally purified rAAV8 vector without increased inflammation and induced anti-vector neutralizing antibodies in a dose-dependent manner. While the RP-rAAV vector method is not intended to replace highly purified viral vector, this simple approach mediates efficient and safe liver transduction *in vivo* and may enable widespread use of this method to facilitate exploratory studies and pilot screening of rAAV constructs in liver research.

## Materials and methods

### Cells and reagents

For AAV production, Human Embryonic Kidney (HEK) 293A cells (Life Technologies) were maintained in Dulbecco’s Modified Eagle Medium (DMEM) containing 4.5 g/L glucose, L-glutamine, and sodium pyruvate supplemented with 10% Fetal Bovine Serum (FBS) and 1% penicillin/streptomycin and 1% L-glutamine (Corning Cellgro; Media Tech). For AAV neutralization assays, Chinese Hamster Ovary (CHO-K1) cells (American Type Culture Collection; ATCC) were grown in Ham’s F12K (Kaighn’s Modified) Medium (Corning Cellgro; Media Tech) supplemented with 10% FBS, 1% penicillin/streptomycin and 1% L-glutamine (Corning Cellgro; Media Tech).

### rAAV production

Iodixanol purified (IP) and rapidly prepared (RP) rAAV vectors were produced in HEK293A cells by an optimized triple transfection protocol as previously described [7]. Briefly, the day before transfection 7.5 x 10^6^ HEK293A cells per T-150 flask (Corning) were plated in DMEM containing 10% FBS and antibiotics. 3.75 *µ*g of endotoxin-free pAAV2/8, 7.5 *µ*g of pAd-Helper, and 3.75 *µ*g of pAAV-CMV-eGFP expressing enhanced green fluorescent protein (eGFP) under the control of a Cytomegalovirus promoter were transfected per T-150 flask using PEI “Max” transfection reagent (Polysciences, Inc.) in DMEM containing 2% FBS. 72 hrs post-transfection cells and culture media were harvested by adding 300 *µ*l of 0.5 *M* EDTA. Total production sizes were thirteen T-150 flasks for the IP method and six T-150 flasks for the RP method.

### rAAV purification by iodixanol gradient-ultracentrifugation

For the iodixanol purification (IP) method, cells and conditioned media were processed separately. Cells were re-suspended in 10 ml of Buffer A (50 m*M* Tris pH 8.5,150 m*M* NaCl) and subjected to three freeze-thaw cycles to release the cell-associated AAV by alternating between ethanol-dry ice and 37 ^o^C water bath. Cell debris was cleared by centrifuging at 3,836 x g for 15 min, followed by treatment with 4 *µ*l of benzonase (Sigma, St. Louis, MO) for 1 hr at 37 ^o^C to digest residual cellular and plasmid DNA. The conditioned cell culture media received 35 m*M* NaCl, was passed through a 0.45 *µ*m filter, and then concentrated approximately 20-fold using a Centricon Plus-70 ultrafilter, MWCO 100,000 (Millipore, Bedford, MA). 10 ml each of processed cell lysate and concentrated supernatant were underlayed in 40 ml Optiseal tubes. This was followed by underlaying four increasingly dense iodixanol step gradients: 3 ml of 15% (w/v) iodixanol, 6 ml of 25% (w/v) iodixanol, 6 ml of 40% (w/v) iodixanol and 4 ml of 58% (w/v) iodixanol. All iodixanol solutions were prepared using OptiPrep^TM^ Density Gradient Medium (Sigma, St. Louis, MO) in 1X Phosphate Buffered Saline (PBS), 1 m*M* Magnesium chloride and 2.5 m*M* potassium chloride. In addition, the 15% (w/v) iodixanol solution contained 0.0075% phenol red and 1 *M* NaCl to destabilize ionic interactions with cell debris, while the 25% (w/v) iodixanol solution contained 0.001% phenol red. The tubes were centrifuged in a 70 Ti rotor in a Beckman Optima XE-90 Ultracentrifuge at 350,333 x *g* (69,000 rpm) for 1 hr 30 min at 18°C. 1 ml fractions were collected by inserting a 16-gauge needle horizontally to the side, 1 cm from the bottom of the tube at the 40%-60% interface. Fractions were subjected to SDS-PAGE and silver stained to assess the purity and visualize AAV8 capsid proteins (VP1, VP2 and VP3). Fractions containing AAV from both gradients were pooled, concentrated and buffer exchanged into an AAV storage buffer (DPBS, 35 m*M* NaCl, 5% glycerol) using an Amicon Ultra-15 (MWCO 100,000) centrifugal filter (Millipore, Bedford, MA).

### rAAV preparation by ultrafiltration

Rapidly prepared (RP) rAAV vector were generated as previously described [7] in the single-flask format, except that six identical rAAV8-CMV-eGFP preparations from individual T-150 tissue culture flasks were processed and ultrafiltered in parallel and then pooled at the end. This allowed us to generate sufficient virus quantities for *in vivo* liver transduction experiments and resulted in significantly reduced hands-on time compared to using one ultrafilter unit sequentially. Briefly, 72 hr after transfection cells and conditioned media were harvested together from each of the six T-150 tissue culture flasks into six 50 ml centrifuge tubes. Tubes were subjected to three freeze-thaw cycles, treated with benzonase (Sigma, St. Louis, MO) to reduce the viscosity, and cell debris were cleared by centrifugation at 3,836 x g for 15 min. The rAAV solution was further concentrated and buffer exchanged into a final AAV storage buffer (DPBS, 35 m*M* NaCl, 5% glycerol) using six Amicon Ultra-15 (MWCO 100,000) centrifugal filter units (Millipore, Bedford, MA) and then pooled.

### Extraction of AAV viral DNA and titration of rAAV8 vectors

rAAV vector titers were determined using quantitative PCR (qPCR). Viral DNA was extracted from RP and IP AAV8 vector preparations with the Maxwell® 16 Viral Total Nucleic Acid Purification Kit (Promega), using an automated Maxwell® 16 instrument configured with low elution volume (LEV) hardware. Briefly, 1 *µ*l of AAV vector sample was digested with 1 U DNase I (Fermentas) in the supplied buffer at 1X in a final volume of 50 *µ*l for 30 min at 37 ^o^C, in order to remove any extra-viral DNA contaminants. DNase I was inactivated by the addition of 5 *µ*l of 50 m*M* EDTA (Fermentas) and heating for 10 min at 65 ^o^C. The samples were then lysed in lysis solution containing 200 *µ*l Maxwell lysis buffer and 20 *µ*l of Proteinase K at 56 ^o^C for 10 min, loaded into the LEV cartridges and processed in the Maxwell instrument. Samples were eluted in 50 *µ*l of nuclease-free water. Both IP and RP AAV8 vectors were tittered using the AAV Inverted Terminal Repeat (ITR) primer-probe set by standard qPCR using Applied Biosystems, 7500 Real Time PCR system as described by Aurnhammer *et al*. [8]. Viral genome titers by ITR assay tend to be significantly higher compared to standard qPCR assays targeting the transgene cassette [9]. In our hands the titer obtained by ITR qPCR is about four fold higher than qPCR assays targeting the transgene, hence a correction factor of 4 was applied to the ITR qPCR titers to account for this difference in titers.

### Infectious titer of rAAV8 vectors

For the infectious titer assay, HEK293A cells were seeded at 1 x 10^5^ cells per well in 24-well plates. The next day, IP and RP rAAV8-CMV-eGFP vectors were serially diluted in DMEM containing 2% FBS supplemented with 1% Penicillin/Streptomycin, 1% L-Glutamine, in the presence or absence of 16.2 m*M* Hoechst 33342 (Thermo Fisher Scientific), and added to the cells [10]. 48 hrs post-infection the number of GFP expressing cells (GFP^+^) was scored in wells that received Hoechst 33342 dye using a Leica microscope at 200X magnification. Wells that did not receive the Hoechst dye had a lower expression with an estimated 10-fold lower GFP^+^ count. Ten random fields were selected and titers in fluorescent focus units (FFU) were calculated based on the formula:

Viral Titer [FFU/ml]=(Average of positive cells/field x 313) x Reciprocal of dilution x 2

### Mouse analysis

All mice were housed at the Animal Facility of Oregon Health & Science University. All studies were approved by the institutional review board. Mouse groups consisting of four to six 129/SvEvTac (129/S; Taconic Biosystems) mice were injected intraperitoneally with 0.75 x 10^11^ vg/mouse, 2.25 x 10^11^ vg/mouse, or 7.50 x 10^11^ vg/mouse of RP-rAAV8-CMV-eGFP or IP-rAAV8-CMV-eGFP vector. Phosphate-buffered saline (PBS) was injected in the seventh mouse group as a negative control. Three weeks post-injection, mice were anesthetized by intraperitoneal injection of mouse cocktail (ketamine 7.5 mg, xylazine 1.5 mg, and acepromazine 0.25 mg/ml). Blood was collected by cardiac puncture and serum was obtained from clotted samples after centrifugation for 10 mins at 10,000 *g* at 4°C. Liver tissue was harvested and stored in liquid nitrogen for assays.

### Western blotting

Mouse liver samples were dounce-homogenized in ice-cold NETT buffer (10 m*M* Tris, pH 7.4, 150 m*M* NaCl, 5 m*M* EDTA, 1% Triton X-100, protease inhibitor cocktail (Bimake.com). Lysates were cleared by centrifugation at 13,000 *g* for 10 minutes at 4°C. Lysate protein concentrations were determined by BCA protein assay (Pierce). Laemmli sample buffer was added to 50 *µ*g of lysate and the samples were incubated at 95°C for 5 minutes. Samples were separated by SDS-PAGE with 15% acrylamide gels. Protein was transferred to nitrocellulose membrane via electrophoresis and blocked with 5% non-fat dry milk in Tris buffered Saline with Tween (TBST). Western blot analysis was performed with anti-GFP primary antibody (Proteintech; 1:5,000 dilution) followed by anti-mouse IgG-HRP conjugate (EMD-Millipore; 1: 10,000 dilution). Bands were detected by chemiluminescence (Supersignal West Pico; Pierce).

### Real-time PCR

Total RNA was isolated from homogenized mouse liver using the Macherey-Nagel Nucleospin RNA isolation kit. cDNA was generated from isolated RNA by Superscript II reverse transcriptase (Life Technologies) with oligo dT primers according to manufacturer’s protocol. Quantitative real-time PCR (qRT-PCR) was performed with SYBR Green master mix (Life Technologies) and an Applied Biosystems ViiA 7. The results are expressed as level relative to *β-*Actin. Target gene primer sequences are listed in **S1 Table**.

### Mouse serum cytokine panel

Nine-week-old 129/SvEvTac male mice (Taconic Biosystems) were injected with either PBS, 7.50 x 10^11^ vg of IP- or RP- rAAV8-CMV-eGFP. Each group contained four mice. Blood samples were obtained immediately prior to injection and 24-hours post-injection via orbital bleed. Mice were sacrificed three weeks post-injection and blood samples were obtained. Serum was isolated as described above and stored at -80°C until use. Serum was analyzed with a Mouse Cytokine Magnetic 20-Plex Panel (Life Technologies) in a Luminex 200 instrument according to manufacturer’s procedures.

### Analysis of AAV8 virus neutralization

#### Collection of mouse sera and treatment

Serum was collected from mice before (pre-injection sera) and after injection (post-injection sera) with saline (PBS), IP-rAAV8-eGFP, and RP-rAAV8-eGFP vectors. All serum samples were heat-inactivated at 56°C for 1 hr and stored as frozen aliquots prior to use.

#### rAAV8 virus neutralization by Luminescence based ID_50_ assay

To measure serum neutralizing activity, CHO-K1 cells were seeded at a density of 5 x 10^4^ cells per well in 96-well plates. The following day, cells were infected with wild-type adenovirus (serotype 5) at an MOI of 118 to help AAV replication. Heat-inactivated mouse sera were serially diluted in duplicate and pre-incubated with an equal volume of 1 x 10^9^ vg of recombinant AAV8 luciferase reporter virus (rAAV8-luc) for 1 hr in 96- well round bottom plates at 37°C. 100 *µ*l of serum-rAAV8-luc mix was then added to the cells previously infected with adenovirus. After 48 hr, Promega Bright-Glo substrate (Promega E2620 -Bright- Glo (TM) Luciferase Assay System) was added to the cells and allowed to incubate at room temperature for 2 mins. 100 *µ*l was then transferred to a 96-well white luminescence plate (Co-star) and luciferase expression was quantitated using the Biotek Synergy Mx luminometer. Pre-injection sera and sera from mice injected with PBS were screened for neutralizing antibody titers at dilutions of 1:50, 1:400 and 1:800. Post-injection sera from mice receiving the rAAV8 vectors were screened using a total of 11 serial two-fold dilutions. The 50% inhibitory dilution (ID_50_) was determined as the serum dilution that resulted in a 50% reduction in relative light units (RLU) compared to wells with the virus only. The ID_50_ or the neutralizing antibody titer at 50% inhibition of virus transduction was calculated using Gen5 software (BioTek) based on the global fitting of the data obtained from each of the 11 dilutions and was based on the 4-parameter formula:

$$y=\frac{a-d}{\left[1+{\left(x/c\right)}^{b}\right]}+d$$

a = (theoretical) response at dilution = 0

b = measure of the slope of the curve at its inflection point

c = value of x at inflection point (ID_50_)

d = (theoretical) response at infinite dilution

x = dilution

y = response (RLU)

As negative control, monkey sera that had been previously validated to be negative for AAV8 neutralization and never achieved 50% neutralization in our assay were used. As positive control, mouse sera that achieved >90% neutralization were used.

### Statistical analysis

For the RT-qPCR experiments, unpaired t-tests with Welch’s correction were used to compare eGFP expression levels between treatment groups at the same dose levels, and one-way ANOVA with Dunnett’s correction was used to compare expression levels of liver inflammatory markers in the treatment groups with the PBS control group. Statistical analysis for the serum cytokine analysis was performed using two-way ANOVA with Bonferroni multiple comparison test comparing three sets of data at each time point. For the neutralizing antibody experiments, unpaired t-tests with Welch’s correction were used to compare ID_50_ titers between treatment groups at the same dose levels. All data were analyzed and plotted using GraphPad Prism version 7 (GraphPad Software, La Jolla, CA).

## Results

### Comparison of iodixanol gradient and rapid rAAV preparation methods

**Fig 1:**
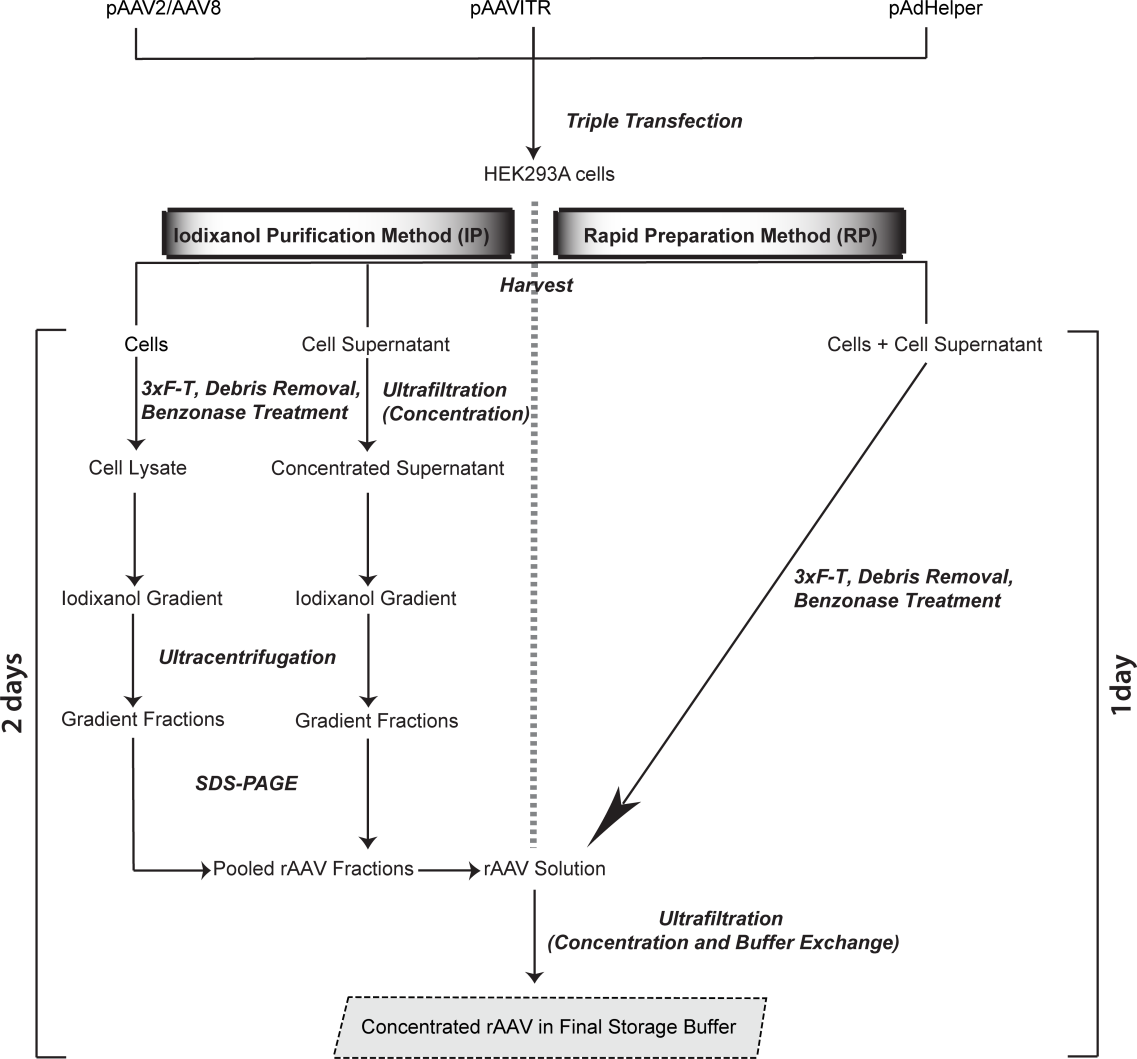
Schematic comparison of the iodixanol purification (IP) and rapid purification (RP) methods. Depicted is a general overview of the steps required to produce, purify, and titer rAAV vectors using the RP and IP methods. F-T = Freeze-Thaw.

A schematic comparison of the iodixanol purification (IP) and the rapid preparation (RP) method is depicted in **Fig 1**. Both approaches use the same optimized PEI-based triple transfection method in HEK293A cells, with the AAV packaging plasmid (pAAV), the transgene plasmid containing viral ITRs (pAAV-ITR), and the helper plasmid (pAdHelper) at a 1:1:2 ratio. The FBS content in the transfection media is reduced from 10% to 2% to facilitate filtration. Upon harvest 72 hrs post-transfection, virus is extracted from cells by three freeze-thaws and cell lysate viscosity is reduced by treatment with benzonase. In the IP method, a total of three separate centrifugations are necessary versus one centrifugation in the RP method **(Fig 1)**. For IP, first the virus contained in the cellular supernatant is concentrated ~100-fold by ultrafiltration before loading onto the iodixanol gradient alongside the virus-containing cell lysate. Second, viral-genome containing rAAV particles are enriched following ultracentrifugation by collecting fractions at the 40–60% iodixanol interface. Fractions are assessed for viral proteins and purity by SDS-PAGE and appropriate fractions are pooled together. Finally, the pooled fractions are concentrated, and buffer exchanged into ~1 ml final volume by ultrafiltration. In our experience, the IP method requires at least two working days **(Fig 1)**: one day to process cells, concentrate cell supernatant, and perform the ultracentrifugation, and another day to select fractions by SDS-PAGE and perform the final concentration and buffer exchange. In contrast, in the RP method the density gradient is bypassed and the two separate ultrafiltration runs are merged into a single step, including parallel-filtration on multiple ultrafilter units to maximize speed and permit simultaneous virus concentration and buffer-exchange. The RP method requires a total hands-on time of about 6 hours post-harvest and thus can be completed in a single day **(Fig 1)**.

### Production of rAAV8 vector by rapid preparation for liver gene transfer in adult mice

In order to evaluate the suitability of our rapid preparation method for AAV-mediated liver gene transfer, we chose AAV8 as it is a widely used serotype that efficiently transduces mouse liver. We reasoned that using the RP method we could quickly produce AAV vectors of serotype 8 and test their ability to enable liver gene transfer studies. To compare the performance of AAV8 vectors prepared by the RP method and the conventional IP method, multiple independent lots of rAAV8 vector expressing enhanced green fluorescent protein (eGFP) under the control of the cytomegalovirus immediate-early promoter (CMV) were prepared by both methods and assayed for physical and infectious titers **(Table 1)**. Due to different resuspension volumes in each virus preparation, the absolute yield of viral genomes (vg) and fluorescent forming units (FFU) rather than relative titers were used to permit more accurate comparisons. The average vg yields of IP-rAAV8-CMV-eGFP (1.6 x 10^13^ vg) were very similar to yields for RP-rAAV8-CMV-eGFP (1.4 x 10^13^ vg), despite using only half the number of tissue culture flasks in the RP method **(Table 1)**. This suggests that RP is more efficient at recovering vg-containing virus particles than IP, and that as expected substantial portions of vg are lost as a result of iodixanol density gradient purification and selective recovery of fractions. Conversely, the infectious particle yields of IP-rAAV8-CMV-eGFP (2.2 x 10^8^ FFU) were about 3-fold higher than yields for RP-rAAV8-CMV-eGFP (8.1 x 10^7^ FFU). This is likely due to the larger IP production size compared to the RP production in this experiment and suggests that infectious particles are not lost in IP. Finally, the RP-based rAAV8 vector retained significant amounts of non-viral proteins, which was expected since the ultrafiltration procedure is solely based on size exclusion (<100,000 MW) and which precludes the direct visualization of AAV viral bands in SDS-PAGE (**S1 Fig**).

**Table 1:** Summary of IP and RP rAAV8 vector productions and titers.

	rAAV8-CMV-eGFP						
	IP-1*	IP-2	IP-3	RP-1*	RP-2	RP-3	
*Final Resuspension Volume (ml)*	1.3	0.63	0.55	0.3	0.22	0.23	
*Viral Genome Titer (vg/mL)*	2.0 x 10^13^	2.8 x 10^13^	6.5 x 10^12^	7.9 x 10^13^	4.9 x 10^13^	3.1 x 10^13^	
*Viral Genome Yield (vg)*	2.6 x 10^13^	1.8 x 10^13^	3.6 x 10^12^	2.4 x 10^13^	1.1 x 10^13^	7.1 x 10^12^	
*Infectious Particle Titer (FFU/mL)*	3.6 x 10^8^	2.5 x 10^8^	7.7 x 10^7^	4.8 x 10^8^	2.3 x 10^8^	2.3 x 10^8^	
*Infectious Particle Yield (FFU)*	4.7 x 10^8^	1.6 x 10^8^	4.2 x 10^7^	1.4 x 10^8^	5.1 x 10^7^	5.3 x 10^7^	
*Mean Viral Genome Yield (vg) ± SD*	1.6 x 10^13^ ± 1.1 x 10^13^			1.4 x 10^13^ ± 8.8 x 10^12^			
*Mean Infectious Particle Yield (FFU) ± SD*	2.2 x 10^8^ ± 2.2 x 10^8^			8.1 x 10^7^ ± 5.1 x 10^7^			

IP and RP production sizes are comprised of 13 and 6 x T-150 flasks, respectively.

* = Viral vectors lots used in mouse experiments

### rAAV8 vector generated by rapid preparation efficiently transduces murine liver *in vivo*

**Fig 2:**
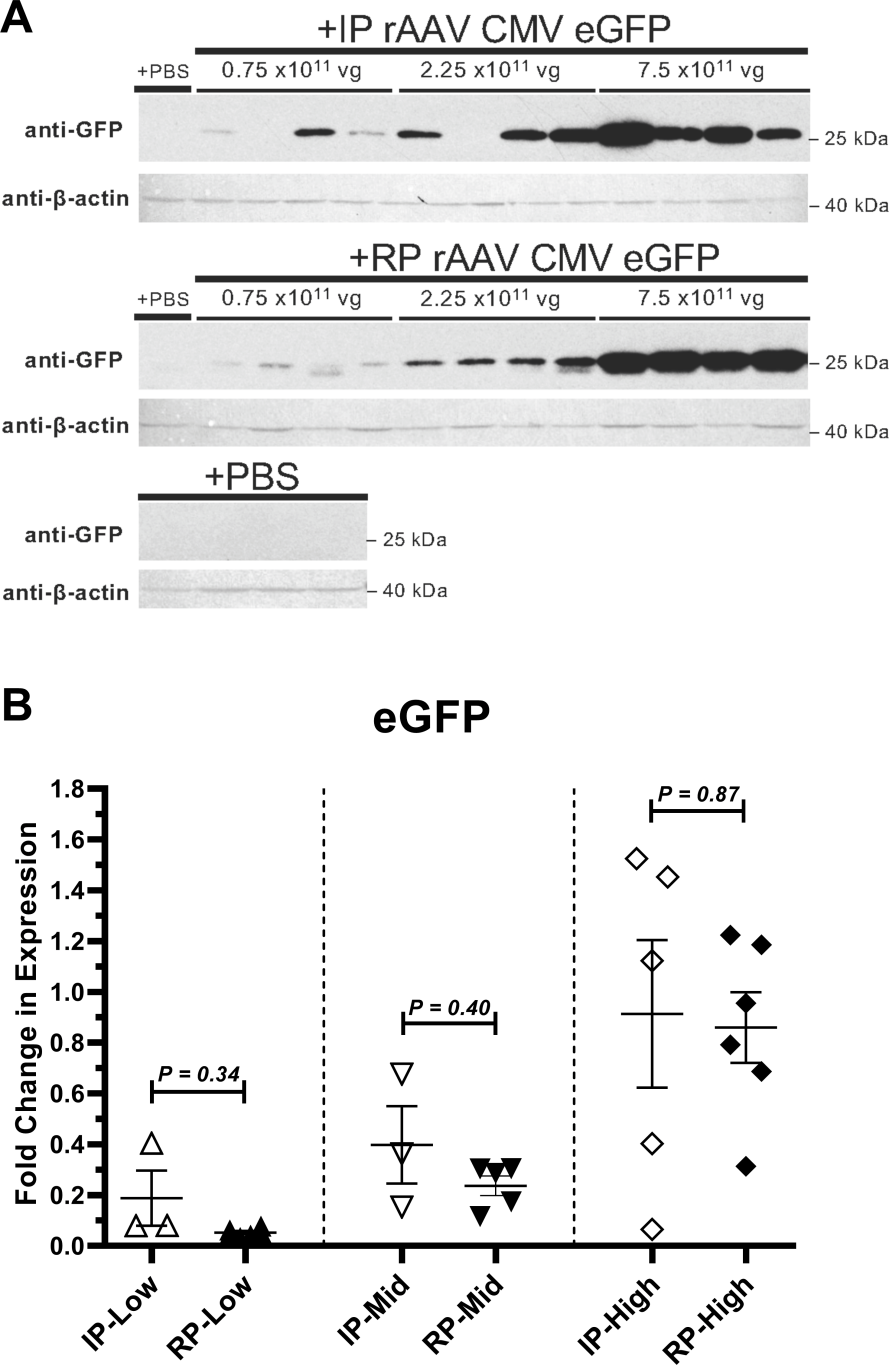
eGFP transgene expression is comparable between IP and RP preparation methods. Liver tissue was obtained from 129/S mice injected intraperitoneally with PBS, IP-rAAV8-CMV-eGFP, or RP-rAAV8-CMV-eGFP. Indicated doses for rAAV8 vectors are 0.75 x 10^11^ vg/mouse (Low), 2.25 x 10^11^ vg/mouse (Mid), and 7.50 x 10^11^ vg/mouse (High). **(A)** Liver tissue lysate was analyzed by anti-GFP immunoblotting. Anti-*β* actin immunoblotting is shown as a loading control. **(B)** Isolated liver RNA was analyzed by quantitative RT-PCR for eGFP transcript level. Expression is represented as relative to the 0.75 x 10^11^ vg RP-rAAV8-CMV-eGFP data set (*β* actin-normalized). Data are represented as scatter plot with mean ± S.E.M. bars (n = 3 to 6 mice). Unpaired t-tests with Welch’s correction were performed to determine p-values.

Since RP-generated rAAV8 vectors performed well in our *in vitro* infectivity assay, we next assessed if these AAV vectors were able to express the eGFP transgene in mouse liver. IP and RP-based rAAV8-CMV-eGFP vectors were intraperitoneally injected in groups of mice (n = 6) with 7.50 x 10^11^ vg/mouse, a dose level results in efficient liver transduction with this serotype and injection route [11] and that we demonstrated to induce supra-physiological transgene expression in the liver [12]. Mice were sacrificed after three weeks and livers were analyzed for eGFP transgene expression and protein. Western blot analysis of four randomly selected mouse liver lysates per group showed strong eGFP bands in all IP and RP rAAV8-CMV-eGFP vector injected animals (**Fig 2A)**. Since eGFP expression with RP-generated vectors was so robust at this dose level, additional cohorts of mice (n = 4 to 6 per group) were injected with lower IP and RP vector doses to determine if less virus could be utilized in this model. Most mice injected with a 3-fold lower dose (2.25 x 10^11^ vg/mouse) of IP or RP-rAAV8-CMV-eGFP vector still showed detectable hepatic eGFP expression. Finally, at the lowest dose (0.75 x 10^11^ vg/mouse), eGFP expression was barely detectable in most mice using Western blot. Animals with undetectable eGFP expression were subsequently found to also lack significant AAV8 neutralizing antibody titers and were therefore considered failed injections and excluded from further analysis (**S2 Table**, starred animals). Further examination of all injection-positive animals by qRT-PCR showed eGFP transcript level increases in the liver as IP and RP virus doses increased **(Fig 2B)**. Importantly, no significant differences in expression were detected between IP and RP preparation methods administered at the same dose levels. Imaging of liver sections showed similarly widespread distribution patterns of eGFP immunofluorescence in the livers of IP and RP-injected animals (**S2 Fig**). We therefore conclude that RP-generated rAAV8 vector efficiently transduces murine liver after intraperitoneal infusion and leads to transgene expression as effectively as the IP method.

### Systemic administration of rapidly prepared rAAV8 vector does not cause liver inflammation or systemic cytokine increase

**Fig 3:**
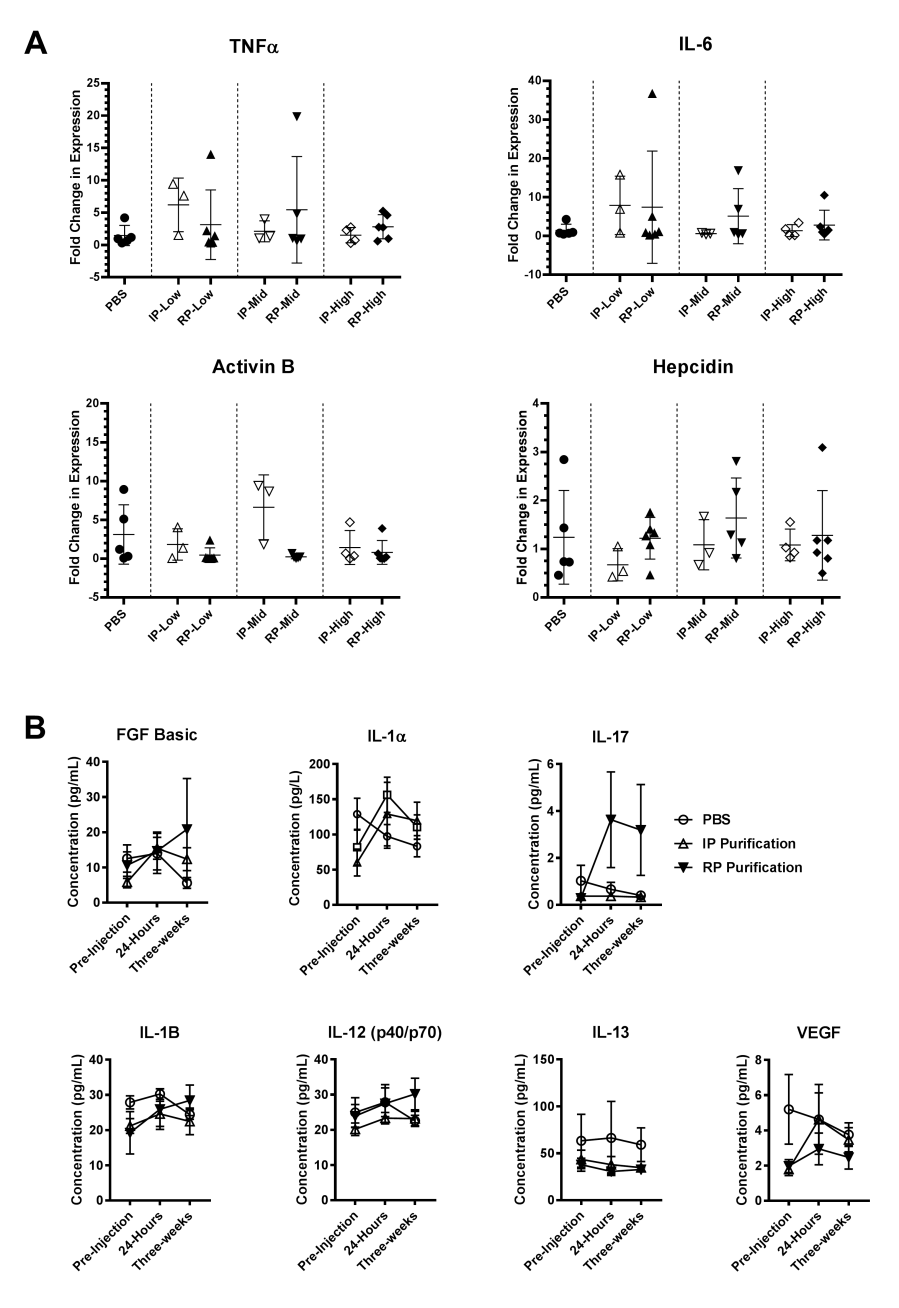
Injection with RP-rAAV8-CMV-eGFP does not induce expression of inflammatory markers in mouse liver or systemic cytokines. 129/S mice were injected intraperitoneally with PBS, IP-rAAV8-CMV-eGFP, or RP-rAAV8-CMV-eGFP. **(A)** Isolated liver RNA was used for quantitative RT-PCR using primers for TNFα, IL-6, Activin B, or Hepcidin. Expression is represented as relative to the PBS data set (*β* actin-normalized). Data are represented as scatter plot with mean ± S.E.M. bars (n = 3 to 6 mice). One-way ANOVA with Dunnett’s correction did not reveal any significant differences in the means of treatment groups compared to the PBS control group. Indicated doses for rAAV8 vectors are 0.75 x 10^11^ vg/mouse (Low), 2.25 x 10^11^ vg/mouse (Mid), or 7.50 x 10^11^ vg/mouse (High). **(B)** Serum cytokine concentrations before and after rAAV injection with 7.50 x 10^11^ vg/mouse were measured by immunoassay. Concentrations units are represented as mean ± S.E.M. (n = 4 mice). Multiple comparison of means using two-way ANOVA with Bonferroni correction did not detect any statistically significant differences between PBS and the treatment groups at each time point. No detectable expression was observed for the cytokines GM-CSF, IFN-γ, IL-2, IL-4, IL-5, IL-6, IL-10, IP-10, KC, MCP-1, MIG, MIP-1α, and TNF-α.

Systemic injection of conventionally purified rAAV8 vector expressing a non-toxic transgene has previously been shown to not induce liver inflammatory responses in host mice [13]. Though the RP method of rAAV preparation showed comparable transgene expression to the IP method, the presence of contaminants in RP vectors could induce inflammation, leading to aberrant physiological responses. To examine the effect of systemic administration of impure RP virus preparations on liver inflammatory responses, mouse liver RNA was examined by qRT-PCR for transcription of the cytokines TNFα, IL-6, and activin B. **Fig 3A** shows that mice that had been injected with either IP or RP rAAV8-CMV-eGFP vectors had similar TNFα, IL-6, and activin B mRNA levels compared to mice injected with PBS, suggesting that the RP-based vector does not induce localized inflammation in murine liver (note that select mice exhibited aberrantly elevated levels of each cytokine, which is likely due to environmental or social factors). We also measured hepcidin mRNA levels as a marker of inflammatory response. Hepcidin is a hormone primarily expressed in hepatocytes that plays a critical role in regulating iron homeostasis. It is induced by IL-6 and activin B making it a sensitive marker of inflammation [14]. Similar to the cytokines, qRT-PCR analysis found no significant upregulation of hepcidin in either IP- or RP-prepared viral injections as compared to the PBS control **(Fig 3A)**. In addition, analysis of liver tissue from multiple mice per treatment group by histopathology **(S3 Fig)** did not reveal any white blood cell infiltration, which would be expected if rAAV vectors induced liver inflammation.

To examine the systemic responses to viral injection, mouse sera were analyzed for the presence of 20 cytokines via a quantitative panel immunoassay pre-injection, at 24 hours, and at 3 weeks post-injection **(Fig 3B).** All cytokines tested showed no statistically significant difference in levels between PBS, IP and RP preparation methods, including IL-6, in agreement with the qRT-PCR results. There was a modest increase in IL-17 after injection of RP-based virus, but did not exceed the normal range (e.g. 0–64 pg/ml for IL-17) [15]. Many cytokines were not present at detectable levels in any of the samples (GM-CSF, IFN-γ, IL-2, IL-4, IL-5, IL-6, IL-10, IP-10, KC, MCP-1, MIG, MIP-1α, TNF-α). Taken together with the liver qRT-PCR and histopathology findings, these observations suggest that RP-rAAV8 vectors expressing transgenes can be administered to mice intraperitoneally without inducing an inflammatory response in liver tissue or provoking a systemic cytokine release.

### Injection of IP and RP rAAV8-CMV-eGFP vectors induce dose-dependent neutralizing antibody responses

**Fig 4:**
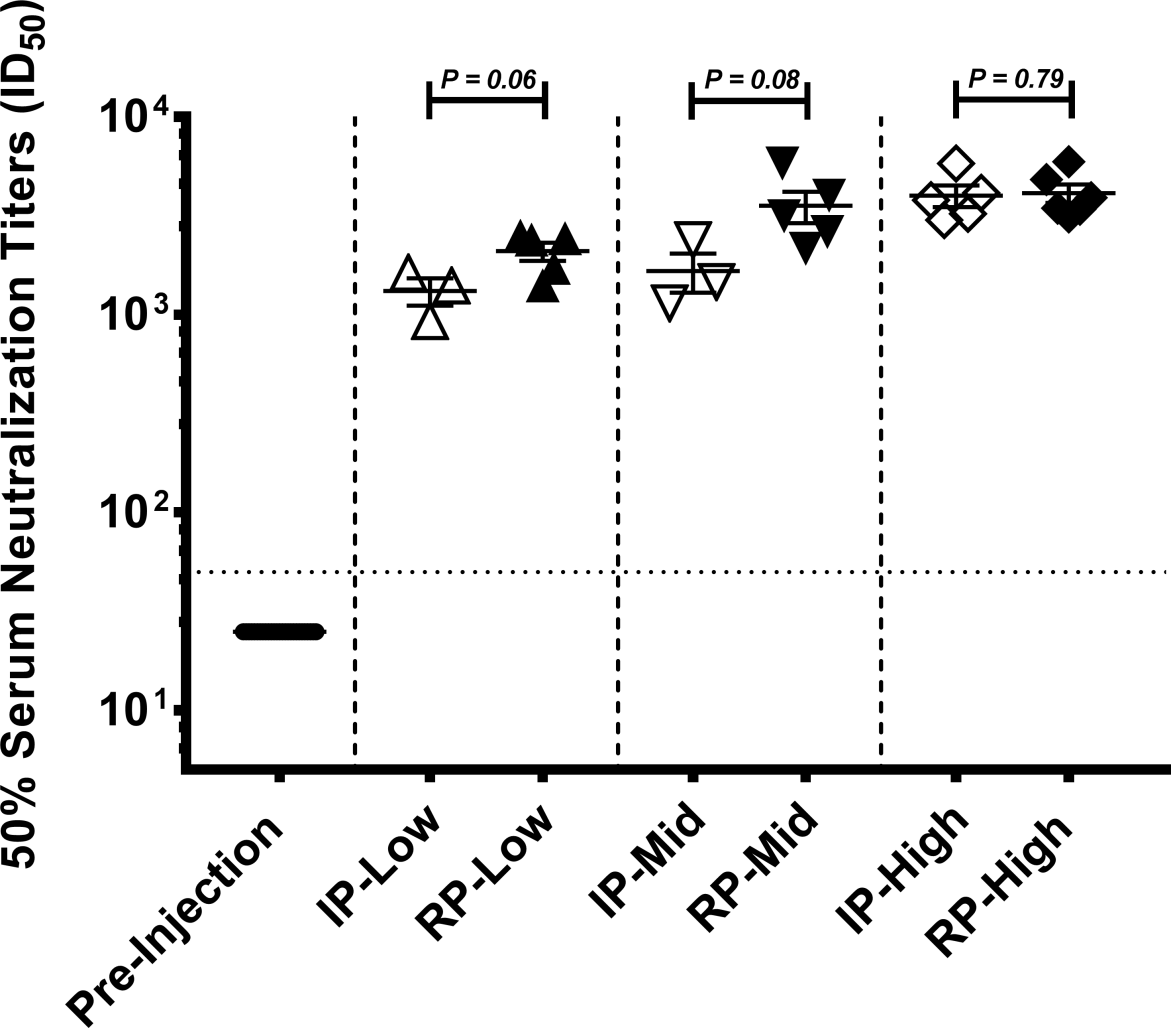
Injection of IP and RP rAAV8-CMV-eGFP vectors induce dose-dependent neutralizing antibody responses. ID_50_ neutralizing antibody assays were carried out on sera of mice that were injected with a 0.75 x 10^11^ vg/mouse (Low), 2.25 x 10^11^ vg/mouse (Mid), or 7.50 x 10^11^ vg/mouse (High) dose of IP or RP rAAV8-CMV-eGFP. Data are represented as scatter plot of neutralizing anti-AAV8 response titers (ID_50_) with mean ± S.E.M. bars (n = 3 to 6 mice). Unpaired t-tests with Welch’s correction were performed to determine p-values. Pre-injection, all mice in the rAAV treatment groups had a negligible anti-AAV8 response (titer <1:50).

Since hepatic and systemic inflammation in response to RP-AAV injection favorably compared to IP-AAV, we next sought to determine if systemic administration of RP-based viral vector would elicit antiviral neutralizing antibody (NAb) responses. Given that RP vector preparations are fairly crude and contain residual cellular and media-derived contaminants, we asked if sera of mice that were injected with RP vector would generate increased virus neutralization titers compared to sera of mice injected with the IP-based vector **(Fig 4)**. Briefly, we performed a luciferase based AAV8 antibody neutralization assay, using both pre-and post-injection sera of mice that were injected with escalating doses of IP or RP-rAAV8-CMV-eGFP vectors. Neutralizing antibody (NAb) titers were defined as the serum dilution at which virus transduction is inhibited >50% compared to the level of virus transduction obtained without serum. For the pre-injection sera, we used three dilutions (1:50, 1:200, and 1:800) to verify that our naïve mice do not have pre-existing neutralizing antibodies to AAV8. As expected, all pre-injection sera and control mice given PBS did not exhibit greater than 50% neutralization activity at the lowest dilution assayed (1:50). More concentrated sera could not be evaluated due to limited serum sample volumes in mice, though any potential low pre-existing antibodies did not appear to effect rAAV8-CMV-eGFP vector transduction as seen by eGFP expression **(Fig 2A–2B)**. For post-injection sera, we performed a full series of 11 dilutions to most accurately determine NAb titers induced by rAAV8 injection. Following injection with the high dose (7.50 x 10^11^ vg/mouse) of rAAV8-CMV-eGFP vectors, the mean neutralization titer for the mouse group injected with the IP vector was 3,999 ± 1,108, and the mean neutralization titer for the mouse group that was injected with the RP vector was 4,106 ± 1,078 **(Fig 4**, P = 0.79). Thus, at the highest vector dose the RP-based vector induced comparable anti-viral NAb responses as the conventionally purified IP vectors. Mouse groups that were injected with a low (0.75 x 10^11^ vg/mouse) dose of IP or RP-vector had mean ID_50_ neutralization titers of 1,318 ± 359 vs 2,093 ± 494 (P = 0.06), and at the moderate (2.25 x 10^11^ vg/mouse) dose had titers of 1,663 ± 645 vs 3,547 ± 1,437 (P = 0.08), respectively **(Fig 4)**. Therefore, both IP and RP-based rAAV8 elicited NAb titers that are directly proportional to the injected virus dose, as is well-established for conventionally purified AAV vectors [16]. Compared to IP-rAAV8, mean NAb titers induced by RP-rAAV8 were about 1.6 to 2-fold higher in the low to mid dose range, whereas titers were similar at the high dose. Though these differences did not reach statistical significance, this suggests that rAAV vectors generated by the rapid ultrafiltration method may induce moderately higher levels of neutralizing antibody responses at low to mid doses, likely due to the presence of production-related residual contaminants. Importantly, even at high RP-vector doses the neutralization response did not eliminate eGFP expression in liver **(Fig 2)**.

## Discussion

Viral vector preparations are generally purified extensively using standard methods in the field before they are applied to a host. In the case of rAAV vectors this has traditionally been accomplished by utilizing density gradients or chromatography-based methods [17, 18]. Even though such purification procedures tend to be tedious and require specialized laboratory instrumentation, a key advantage is that the majority of production related impurities, i.e. mainly cellular and media components, are excluded from the final vector product. However, even pure rAAV vector stocks prepared with established methods can still contain residual impurities that can negatively impact viral transduction [19]. For example, rAAV vectors purified by standard CsCl density gradients can contain significant levels of proteins and nucleic acids [20]. Moreover, in some studies CsCl-purified rAAV vectors have shown significant cytotoxicity in cell culture and demonstrated altered cell targeting properties *in vivo* compared to the same rAAV vectors purified via iodixanol gradient [21].

While *in vitro* experiments are sometimes conducted with crude viral lysates, *in vivo* administration of significant amounts of debris present in unpurified virus stocks were assumed to have toxic and immunogenic effects on the host. Our work in this study demonstrates that systemic injection of minimally processed rAAV vector is safe and efficiently transduces the liver, and thus RP-derived rAAV vectors are useful tools for pilot liver gene transfer studies. Specifically, we have shown that such rAAV8 vector stocks can be rapidly prepared using a simple ultrafiltration technique, which we call rapid preparation (RP), and which does not include density gradient purification. Intraperitoneal administration of RP-based rAAV8 vector encoding eGFP led to efficient liver transduction and expression of eGFP, which was comparable to the transduction efficiencies observed with iodixanol purified (IP) rAAV8 vector over a range of administered vector doses that are commonly used for AAV-mediated transduction in mice. Importantly, no significant liver inflammation, infiltration, or systemic cytokine secretion could be detected in response to either RP or IP-based rAAV8 vector injection. Neutralizing antibody responses to both vector preparations were comparable at the highest dose, and up to two-fold higher in the low and mid RP dose groups. Together, these data indicate that residual contaminants present in RP viral vector preparations do not interfere with transduction and do not enhance inflammation or markedly exacerbate humoral immune responses in the host.

Previously, we described a cost-effective, rapid preparation (RP) method to produce rAAV1 vector for direct fetal injection *in vivo* into the developing mouse inner ear [7]. The RP method involves ultrafiltration to directly concentrate and buffer-exchange rAAV1 particles harvested from both cell lysates and culture supernatants following triple transfection. Although rAAV preparations obtained by the RP method still contain fairly high amounts of cellular contaminants, when injected directly into the developing mouse inner ear, RP-rAAV1 vectors did not induce any detectable toxicity or morphological changes in the fetal setting [7]. Another significant advantage of the RP method is that many different rAAV can be produced and prepared simultaneously on a small scale (“AAV minipreps”), and we have used this approach to efficiently screen expression of different rAAV constructs in inner ear pilot studies. The injections were done locally in prenatal mice, which did not have a fully developed immune system. Our present results indicate that injection of up to 7.50 x 10^11^ vg RP-rAAV8 vector intraperitoneally in adult mice does not result in either a liver or systemic inflammatory response. Doses in this range are commonly used thus increasing the feasibility of a more general application of RP-produced rAAV vectors in exploratory gene therapy research.

Since we have observed a very similar *in vivo* performance of RP and IP-based rAAV8 vectors in our studies, this raises the question whether virus stocks prepared by rapid ultrafiltration differ significantly from those purified by iodixanol gradient. There has been a longstanding and reasonable assumption that production-related impurities and empty viral capsids that are present in unpurified and partially purified rAAV stocks will interfere with viral transduction and increase host inflammatory and immune responses. We explored this question in the current study, since we could not find any published evidence supporting this notion. Cellular factors retained in AAV crude lysates, specifically constituents in 293-derived producer cell extracts, actually enhance rAAV vector transduction [22]. RP-rAAV cannot be considered an entirely crude lysate as ultrafiltration is expected to remove the majority of small proteins and nucleic acids <100,000 MW. However, rAAV prepared by the RP method displays many non-viral (i.e. HEK293A cell and FBS-derived) protein bands in SDS-PAGE (**S1 Fig**). Removal by the filter of small inhibitory contaminants could explain the robust *in vivo* transgene expression observed with RP-rAAV in our study. In addition, small transgene products commonly expressed from rAAV vectors, such as the 27-kDa eGFP in our vector, are filtered out. Thus, RP-based rAAV vectors are not expected to suffer from the problem of pseudo-transduction (i.e. non-viral transfer of contaminating transgene product to target cells) as has been observed with crude virus lysates [23, 24], and we did not observe any signs of pseudo-transduction by RP-based virus *in vitro* (comparable infectious titers to IP-based virus, **Table 1**) or *in vivo* (eGFP expression was determined 3 weeks post-injection, suggesting it is not due to transient protein transfer, **Fig 2**). Future studies will be needed to better understand the effects of different production-related contaminants on the host. For example, the toxicity and *in vivo* performance of fully crude rAAV will need to be tested. Conversely, RP-AAV vector purity and ultrafiltration speed could be further improved by completely omitting FBS supplementation from the media during production, provided that acceptable virus yields can be obtained.

Empty virus capsids have long been postulated to interfere with virus transduction, since they compete for target cell receptor binding sites but lack the transgene-containing virus genome and as their removal can increase transduction efficiency [20, 25]. They can also increase the antigenic load in the host, leading to inflammation [26] and greater immune responses [27–29]. A perceived advantage of using CsCl in density gradient purification is its ability to significantly reduce the empty particle content of AAV preparations [26]. Interestingly, recent work suggests potential advantages of including empty viral capsids as “capsid decoys” to neutralize anti-AAV antibody responses [30]. Since the RP method does not separate virus particles by density, it will not remove empty viral capsids. However, neither does iodixanol purification, as IP-based AAV vector preparations have been shown to contain a significant proportion of empty capsids [31]. Since there are empty viral capsids present in both preparations, they would be expected to affect transduction, inflammation, and NAb responses similarly. This is also what we observed, with the exception of up to two-fold higher NAb responses at the low to mid RP rAAV8 doses. Based on this it is possible that RP-based vectors retain a greater proportion of empty viral capsids than IP-based vectors.

rAAV vectors have become a key viral gene transfer platform, and thus significant efforts have been dedicated to improve virus purification methods in recent years, particularly scalable and serotype-specific chromatography-based approaches that can be translated to the needs of vector production for clinical trials [32]. Traditional density gradient-based methods favored for pre-clinical and pilot studies are generally serotype-independent, but still require a significant time investment, which has only been increased by modifications to these methods [21, 33]. Other methods that do not require ultracentrifugation through density gradients have been recently developed, though these still need multiple virus spin and precipitation steps and require the use of toxic reagents [34], tangential flow filtration equipment [35], or an ultracentrifuge [36]. The simple and modular AAV ultrafiltration approach applied here, while not removing contaminants >100,000 MW, nevertheless generates viral vector preparations capable of safe and efficient transduction of mouse liver *in vivo*. We had initially established the RP method to enable production of rAAV1 vectors for fetal gene transfer to the mouse inner ear [7]. Since targeting this minute tissue involves microinjection of nanoliter amounts of viral vector, RP-AAV1 vector preparations were generated at small scale (one T-150 flask). In the current study that involved systemic gene delivery targeting adult mouse liver, the RP method was used to purify AAV serotype 8 vectors and was modularly sized up to six T-150 flasks (see Methods), in order to generate sufficient amounts of RP-based rAAV8 vector for *in vivo* mouse studies. Since in RP the virus material from individual T-150 production flasks is processed and ultrafiltered individually and then combined at the end, the method is modular and therefore easily customizable from one to several T-150 flasks. Together, the RP method fills an important unaddressed niche for small- to medium-size rAAV preparation that is both rapid and serotype-independent. The harvest and partial purification of vectors takes a single day and yields sufficient quantities of viral inoculum for small *in vivo* studies. The speed of vector preparation could be further increased by additional refinements to our protocol, such as by utilizing producer cells grown in suspension that can be more easily grown and manipulated, or by lysing cells using a sonicator or microfluidizer, although we have designed the current protocol to be conducted in a standard tissue culture laboratory without needing access to specialized equipment or know-how. We have used this approach to simultaneously generate a dozen “minipreps” of distinct rAAV vector constructs for rapid and inexpensive screening of small AAV libraries *in vivo* [7]. In the same way, while this simple procedure is not meant to replace stringent purification methods where high virus purity is required, RP is useful for generating small to medium amounts of semi-crude AAV vector material for pilot experiments, enabling the rapid and cost-efficient testing of hypotheses.

## Supporting information

S1 Materials and methods (DOCX)

S1 Table Primers used in qRT-PCR analysis. (DOCX)

S2 Table Individual mouse liver eGFP expression levels and serum AAV8 neutralizing antibody titers. (DOCX)

S1 Fig SDS-PAGE of IP and RP prepared rAAV8-CMV-eGFP vectors. IP and RP-based rAAV8 CMV-eGFP vectors were separated on a reducing SDS-PAGE gel and stained with SYPRO^®^ Ruby. AAV viral proteins VP1 (89 kDa), VP2 (75 kDa), and VP3 (64 kDa) are indicated by white asterisks. Lane 1, Low Molecular Weight Protein Standard; lanes 2−4, contain 4 x 10^10^ vg, 6 x 10^10^ vg and 8 x 10^10^ vg of IP-rAAV8-CMV-eGFP vector, respectively; lanes 5−10 contain 5 x 10^8^ vg, 1 x 10^9^ vg, 2 x 10^9^ vg, 4 x 10^9^ vg, 5 x 10^9^ vg and 1 x 10^10^ vg of RP-rAAV8-CMV-eGFP vectors, respectively. Various non-AAV protein bands are visible in the RP-based vector preparation, which stem from residual production-related impurities and largely obscure the AAV VPs. The bright band at ~66 kDa is consistent with albumin, a major constituent of the fetal bovine serum (FBS) used in the vector production process.
(TIF)

S2 Fig Representative images of liver tissue sections showing similar eGFP distribution in livers of mice injected with IP and RP rAAV8 vectors. 3 weeks after injection with PBS, IP-rAAV8-CMV-eGFP (7.5 x 10^11^ vg/mouse), or RP-rAAV8-CMV-eGFP (7.5 x 10^11^ vg/mouse), livers were harvested from one mouse (PBS group) or two mice (IP High and RP High groups) per group. 16µm thick liver tissue sections were mounted on slides, nuclei stained with Hoechst 33258 (blue), and imaged with an Olympus VS110 slide scanner using a 20x air objective with a XM10 camera to capture DAPI (blue) and eGFP fluorescence (green). Representative sections shown are from animals PBS 5 (A), IP High 5 (B), IP High 6 (C), RP High 5 (D), and RP High 6 (E). Both IP and RP prepared vectors display comparable native eGFP (green) brightness and general distribution across the liver. Individual fluctuations in the number of eGFP positive cells are consistent with the variation in liver eGFP mRNA expression levels as listed in S2 Table. Animal IP High 6 (C) showed no visible eGFP expression, consistent with having only background levels of eGFP mRNA expression.
(TIF)

S3 Fig Hematoxylin and eosin stain of mouse livers. Mice were injected with PBS, IP-purified rAAV8-CMV-eGFP (7.5 x 10^11^ vg/mouse), or RP-rAAV8-CMV-eGFP (7.5 x 10^11^ vg/mouse). After 3 weeks, livers from four mice per group were harvested and stained with hematoxylin and eosin. All images in a column belong to the indicated treatment group and are from individual mice from that group. No evidence of immune cell infiltrates was seen in any of the livers.
(TIF)
