# Supplementary material for: Ultrafiltered recombinant AAV8 vector can be safely administered in vivo and efficiently transduces liver
Source: PLoS One. 2018 Apr 5;13(4):e0194728. doi: 10.1371/journal.pone.0194728 (PMC5886455; doi:10.1371/journal.pone.0194728)
Supplement: S1 Materials and methods — (DOCX) [file pone.0194728.s001.docx]

**S1 Materials and methods**

**SDS-PAGE of rAAV8 vector preparations**

IP-rAAV8-CMV-eGFP and RP-rAAV8-CMV-eGFP samples were prepared in 2X Laemmli sample buffer (Bio-Rad) containing 350 mM Dithiothreitol (DTT) reducing agent and boiled for 5 mins at 95°C. Samples were loaded onto a 10% Mini-PROTEAN® TGXTM Precast Gel (Bio-Rad) and run in 1X Tris/Glycine/SDS running buffer (Bio-Rad) at 150V for 90 mins. Gel was stained using SYPRO® Ruby (Molecular Probes, Invitrogen, ThermoFisher Scientific) according to the manufacturer’s instructions for the rapid protocol. Briefly, the gel was rinsed with water, fixed in 50% methanol, 7% acetic acid twice for 15mins, then stained with SYPRO® Ruby for 30 mins, followed by a final wash with 10% methanol and 7% acetic acid for 30 mins. AAV Viral Proteins (VP) were visualized on the gel using a Molecular Imager Gel DocTM XR+ (Bio-Rad) and imaged using Image Lab Software 5.2.1 (Bio- Rad). Molecular weights of protein bands were based on the Low Molecular Weight Protein Standard (1:1000) (Bio-Rad).

**Liver tissue preparation, staining, and immunofluorescence**

3 weeks after injection with PBS, IP or RP- rAAV8-CMV-eGFP mice were anesthetized with 4% isoflurane and transcardially perfused with 10ml of saline to clear blood followed by 10ml of 4% paraformaldehyde (PFA). Liver tissue was harvested and immersed in 4% PFA for 1h. Liver samples for histopathology were given to the OHSU Histopathology Shared Resource in the Knight Cancer center for processing, sectioning, and hematoxylin and eosin staining. Liver samples destined for eGFP microscopy were transferred to 1% PFA and stored at 4°C. Prior to embedding, liver tissue was placed in 30% sucrose for 24 hrs, then placed in standard cryo-molds (TissueTek 4557) containing the embedding media O.C.T. (TissueTek) and snap frozen in a cold isopentane bath. The frozen liver tissue was sectioned using a Leica CM 3050S cryostat into 16µm sections and mounted onto Super Frost Plus glass slides (Fisher). Slides were washed with PBS, counter-stained with Hoechst 33258 nuclear stain (1: 10,000 dilution; Invitrogen), mounted with Slowfade Gold Antifade mounting media (Life Technologies), and covered with Fisherbrand Microscope Cover Glass (Fisher).

**Microscopic imaging**

Images were obtained using the Olympus VS110 slide scanner controlled by the VS-ASW FL 2.7 (Build 11043) using a 20x air objective (UPLSAPO 20x / NA 0.75) with a XM10 camera (Olympus Soft Imaging Solutions). Multiple, continuous fields of view on the XYZ planes were imaged throughout the entire liver section for each group, (7 Z-steps with a 1.34 µm system optimized Z spacing for each field of view) were obtained. The fluorophores were excited using an X-Cite exacte (Excelitas Technologies) with a 200W Mercury Arc Lamp. The DAPI fluorescence from the nucleus was captured with a 440 nm emission wavelength SEDAT filter cubes at a constant 330 ms exposure time. The eGFP fluorescence was captured with a 560 nm emission wavelength SEDAT filter cubes at a constant 55 ms exposure time. The settings were kept constant for all images.

**Post-processing of images**

The XYZ-stacks obtained were processed using the Olympus CellSens Dimension Desktop software (Version 1.17, Olympus Soft Imaging Solutions). The stacks were processed with Extended Focal Imaging (EFI) processing. The output image is a single plane focused image. The single plane images thus obtained were cropped as new images with a constant area for each group. No other image alterations were done. Cropped images were saved as TIFF files and Figure panels were prepared using the ScientiFig plugin in the ImageJ software.
