# Supplementary material for: Ultrafiltered recombinant AAV8 vector can be safely administered in vivo and efficiently transduces liver
Source: PLoS One. 2018 Apr 5;13(4):e0194728. doi: 10.1371/journal.pone.0194728 (PMC5886455; doi:10.1371/journal.pone.0194728)
Supplement: S1 Table — (DOCX) [file pone.0194728.s002.docx]

**S1 Table**. **Primers used in qRT-PCR analysis.**

| **Mouse gene** | **Forward Primer** | **Reverse Primer** |
| --- | --- | --- |
| *Activin B* | 5'-TCAGCTTTGCAGAGACAG-3' | 5'-GAAGAAGTACAGGCGGAC-3' |
| *β-Actin* | 5'-CTGCCTGACGGCCAGGT-3' | 5'-TGGATGCCACAGGATTCCAT-3' |
| *eGFP* | 5'-CACCCACGTGACCACCCTTAC-3' | 5'-GGATGTTGCAGTCCTCCCTG-3' |
| *Hepcidin* | 5'-CACCAACTTCCCCATCTGCATCTT-3' | 5'-GAGGGGCTGCAGGGGTGTAGAG-3' |
| *IL-6* | 5'-TTCCATCCAGTTGCCTTCTTG-3' | 5'-TTGGGAGTGGTATCCTCTGTGA-3' |
| *TNFα* | 5’-CGTCAGCCGATTTGCTATCT-3’ | 5’-CGGACTCCGCAAAGTCTAAG-3’ |
