# Supplementary material for: Ultrafiltered recombinant AAV8 vector can be safely administered in vivo and efficiently transduces liver
Source: PLoS One. 2018 Apr 5;13(4):e0194728. doi: 10.1371/journal.pone.0194728 (PMC5886455; doi:10.1371/journal.pone.0194728)
Supplement: S2 Table — (DOCX) [file pone.0194728.s003.docx]

**S2 Table. Individual mouse liver eGFP expression levels and serum AAV8 neutralizing antibody titers.**

| **Animal Group and Number** | **eGFP Liver mRNA [Fold Change in Expression]** | **Neutralizing Antibody Titer [ID_50_]** |
| --- | --- | --- |
| *PBS 1* | 0.00 | <1:50 |
| *PBS 2* | 0.00 | <1:50 |
| *PBS 3* | 0.00 | <1:50 |
| *PBS 4* | 0.00 | <1:50 |
| *PBS 5* | 0.01 | <1:50 |
| *IP-Low 1* | 0.08 | 924 |
| *IP-Low 2** | 0.01 | <1:50 |
| *IP-Low 3* | 0.08 | 1402 |
| *IP-Low 4* | 0.41 | 1628 |
| *IP-Mid 1* | 0.15 | 2384 |
| *IP-Mid 2** | 0.09 | 95 |
| *IP-Mid 3* | 0.36 | 1142 |
| *IP-Mid 4* | 0.68 | 1463 |
| *IP-High 1* | 0.06 | 5815 |
| *IP-High 2* | 1.12 | 3235 |
| *IP-High 3* | 1.52 | 3013 |
| *IP-High 4* | 0.40 | 3798 |
| *IP-High 5* | 1.45 | 4135 |
| *IP-High 6** | 0.02 | <1:50 |
| *RP-Low 1* | 0.03 | 1410 |
| *RP-Low 2* | 0.08 | 2433 |
| *RP-Low 3* | 0.06 | 1726 |
| *RP-Low 4* | 0.04 | 2392 |
| *RP-Low 5** | 0.05 | <1:50 |
| *RP-Low 6* | 0.05 | 2504 |
| *RP-Mid 1* | 0.30 | 5806 |
| *RP-Mid 2* | 0.17 | 2617 |
| *RP-Mid 3* | 0.30 | 2159 |
| *RP-Mid 4* | 0.29 | 4004 |
| *RP-Mid 5* | 0.12 | 3151 |
| *RP-Mid 6** | 0.10 | <1:50 |
| *RP-High 1* | 1.22 | 3450 |
| *RP-High 2* | 0.96 | 3114 |
| *RP-High 3* | 0.69 | 4823 |
| *RP-High 4* | 1.19 | 5939 |
| *RP-High 5* | 0.31 | 3900 |
| *RP-High 6* | 0.79 | 3410 |

*Animals excluded from further analysis due to lack of significant AAV8 neutralizing antibody titer

eGFP mRNA expression is represented as relative to the 7.50 x 10^11^ vg IP-rAAV8-CMV-eGFP data set (*β* actin-normalized) and are plotted in Figure 2B.

Neutralizing antibody titers to AAV8 are represented as ID50 and are plotted in Figure 4.
