# Supplementary figures and images for: Ultrafiltered recombinant AAV8 vector can be safely administered in vivo and efficiently transduces liver
Source: PLoS One. 2018 Apr 5;13(4):e0194728. doi: 10.1371/journal.pone.0194728 (PMC5886455; doi:10.1371/journal.pone.0194728)

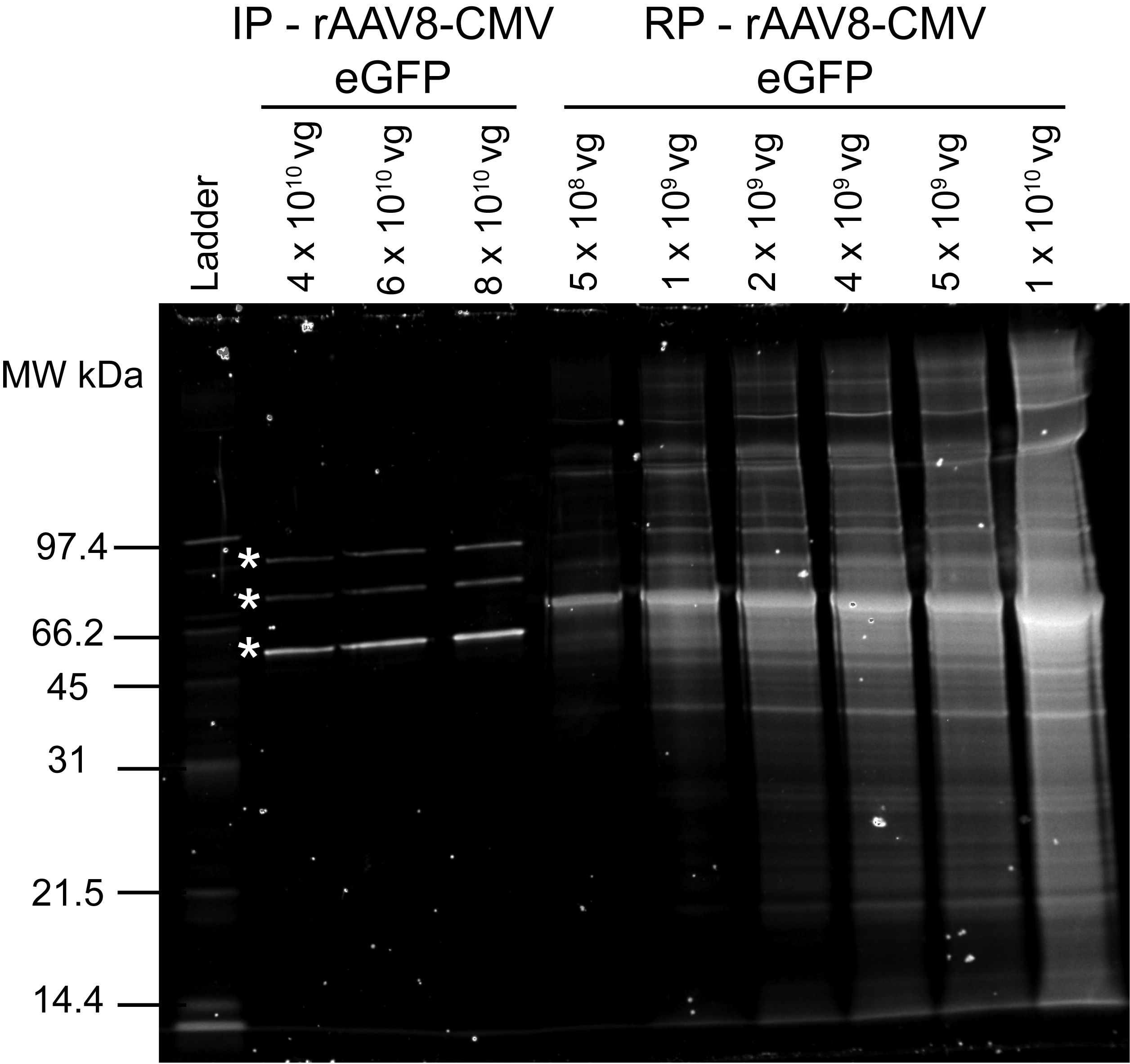

Supplement: S1 Fig — IP and RP-based rAAV8 CMV-eGFP vectors were separated on a reducing SDS-PAGE gel and stained with SYPRO® Ruby. AAV viral proteins VP1 (89 kDa), VP2 (75 kDa), and VP3 (64 kDa) are indicated by white asterisks. Lane 1, Low Molecular Weight Protein Standard; lanes 2−4, contain 4 x 10^10^ vg, 6 x 10^10^ vg and 8 x 10^10^ vg of IP-rAAV8-CMV-eGFP vector, respectively; lanes 5−10 contain 5 x 10^8^ vg, 1 x 10^9^ vg, 2 x 10^9^ vg, 4 x 10^9^ vg, 5 x 10^9^ vg and 1 x 10^10^ vg of RP-rAAV8-CMV-eGFP vectors, respectively. Various non-AAV protein bands are visible in the RP-based vector preparation, which stem from residual production-related impurities and largely obscure the AAV VPs. The bright band at ~66 kDa is consistent with albumin, a major constituent of the fetal bovine serum (FBS) used in the vector production process. (TIF) [file pone.0194728.s004.tif]

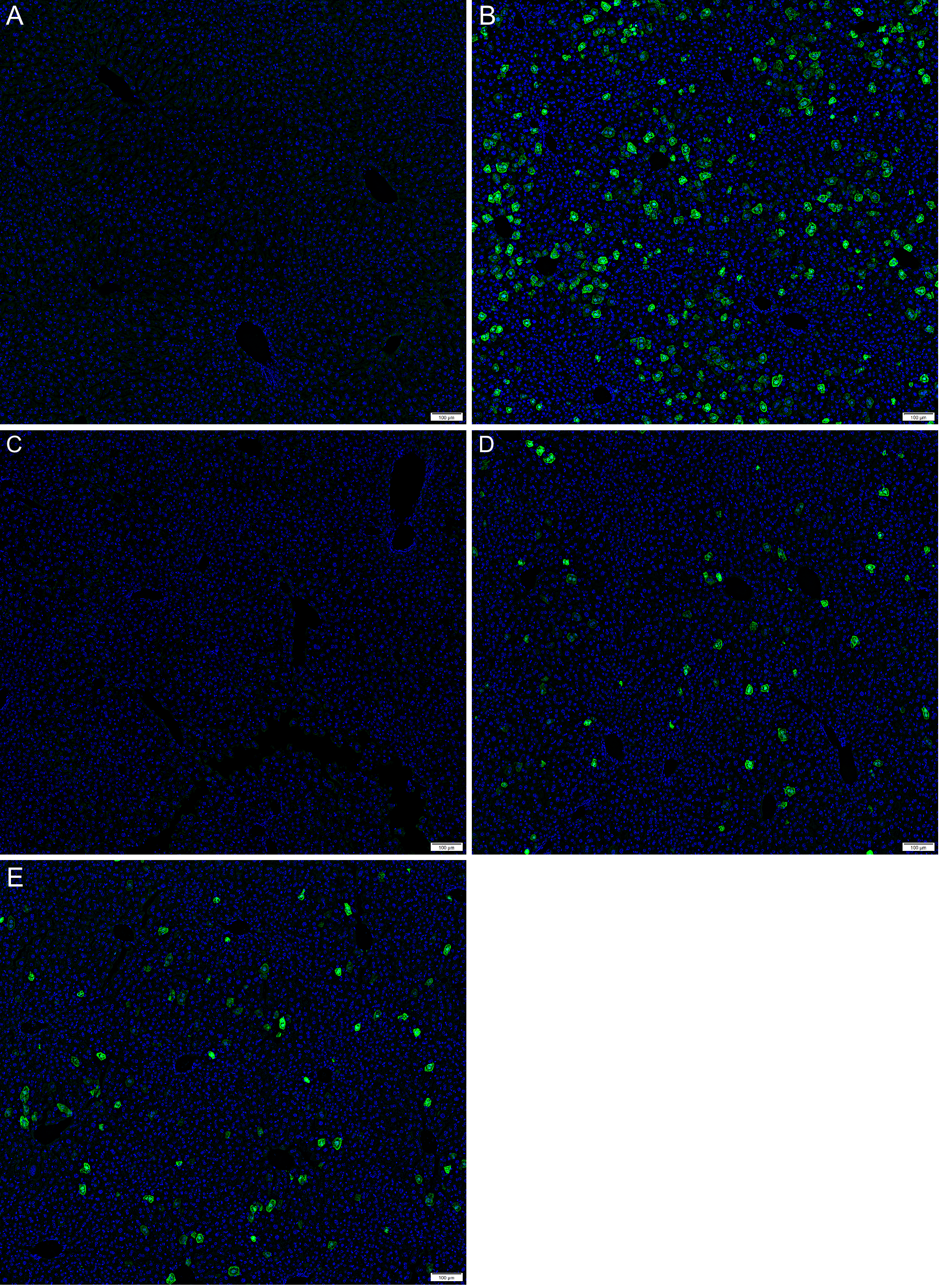

Supplement: S2 Fig — 3 weeks after injection with PBS, IP-rAAV8-CMV-eGFP (7.5 x 10^11^ vg/mouse), or RP-rAAV8-CMV-eGFP (7.5 x 10^11^ vg/mouse), livers were harvested from one mouse (PBS group) or two mice (IP High and RP High groups) per group. 16µm thick liver tissue sections were mounted on slides, nuclei stained with Hoechst 33258 (blue), and imaged with an Olympus VS110 slide scanner using a 20x air objective with a XM10 camera to capture DAPI (blue) and eGFP fluorescence (green). Representative sections shown are from animals PBS 5 (A), IP High 5 (B), IP High 6 (C), RP High 5 (D), and RP High 6 (E). Both IP and RP prepared vectors display comparable native eGFP (green) brightness and general distribution across the liver. Individual fluctuations in the number of eGFP positive cells are consistent with the variation in liver eGFP mRNA expression levels as listed in S2 Table. Animal IP High 6 (C) showed no visible eGFP expression, consistent with having only background levels of eGFP mRNA expression. (TIF) [file pone.0194728.s005.tif]

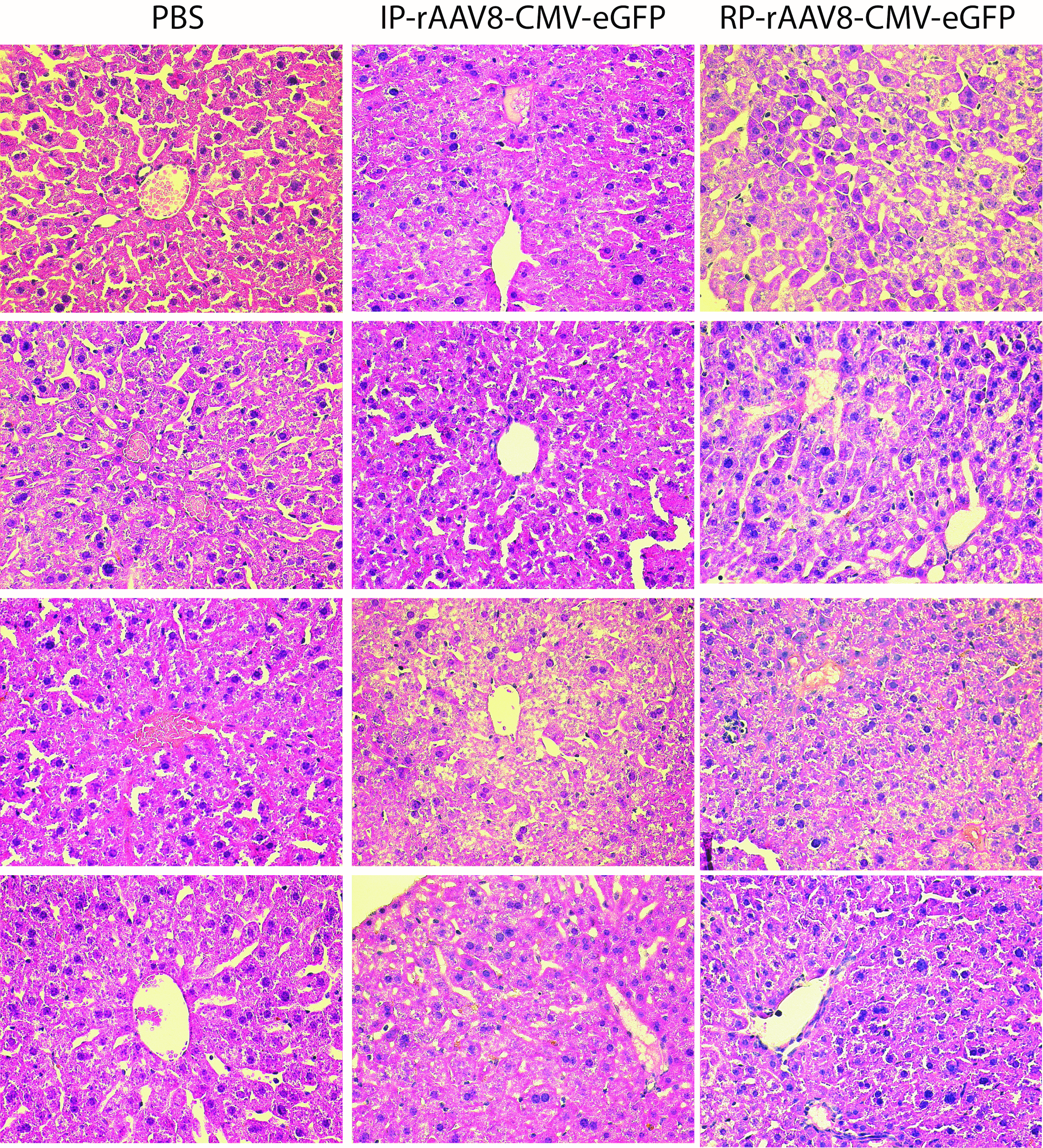

Supplement: S3 Fig — Mice were injected with PBS, IP-purified rAAV8-CMV-eGFP (7.5 x 10^11^ vg/mouse), or RP-rAAV8-CMV-eGFP (7.5 x 10^11^ vg/mouse). After 3 weeks, livers from four mice per group were harvested and stained with hematoxylin and eosin. All images in a column belong to the indicated treatment group and are from individual mice from that group. No evidence of immune cell infiltrates was seen in any of the livers. (TIF) [file pone.0194728.s006.tif]
